# Influence of N-Acetylglucosamine and Melatonin Interaction in Modeling the Photosynthetic Component and Metabolomics of Cucumber under Salinity Stress

**DOI:** 10.3390/ijms25052844

**Published:** 2024-02-29

**Authors:** Sang-Mo Kang, Arjun Adhikari, Eun-Hae Kwon, Ho-Jun Gam, Jin Ryeol Jeon, Ji-In Woo, In-Jung Lee

**Affiliations:** 1 Department of Applied Biosciences, Kyungpook National University, Daegu 41566, Republic of Korea; kmoya@hanmail.net (S.-M.K.); arjun@knu.ac.kr (A.A.); eunhae.kwon1@gmail.com (E.-H.K.); jeff4237@gmail.com (H.-J.G.); 98micael10@naver.com (J.R.J.); wjxsj99@naver.com (J.-I.W.); 2 Institute of Agricultural Science and Technology, Kyungpook National University, Daegu 41566, Republic of Korea

**Keywords:** oxidative stress, chlorophyll fluorescence, environment, biostimulants, minerals

## Abstract

The application of N-acetylglucosamine (GlcNAc) and melatonin (Mel) in agriculture could be a promising avenue for improving crop resilience and productivity, especially under challenging environmental conditions. In the current study, we treated the cucumber plant with GlcNAc and Mel solely and combinedly under salt stress (150 mM) then studied photosynthetic attributes using the transient OJIP fluorescence method. The results showed that the combination of GlcNAc × Mel significantly improved the plant morphological attributes, such as root and shoot biomass, and also improved chlorophyll and photosynthetic components. The mineral elements such as K, Mg, Ca, and P were significantly elevated, whereas a lower influx of Na was observed in GlcNAc × Mel treated cucumber shoots. A significant reduction in abscisic acid was observed, which was validated by the reduction in proline content and the increase in stomatal conductance (Gs), transpiration rate (E), and substomatal CO_2_ concentration (Ci). Furthermore, the activities of antioxidants such as polyphenol and flavonoid were considerably improved, resulting in a decrease in SOD and CAT with GlcNAc × Mel treatment. In addition, GlcNAc × Mel treatment dropped levels of the toxic radical Malondialdehyde (MDA) and elevated amino acids in cucumber shoots. These findings suggest that the combination of GlcNAc × Mel could be an effective elicitor for modeling plant metabolism to confer stress tolerance in crops.

## 1. Introduction

Extreme environmental stresses have long been a significant threat to crop productivity and sustainable agriculture [1]. Salinity stress is one of the major environmental stressors that negatively affects global crop productivity [2]. Salinity occurs through natural or human-induced processes that result in the accumulation of dissolved salts (anions—Cl^−^, SO_4_^2−^, and HCO_3_, and cations—Na^+^, Mg^2+^, and Ca^2+^) in the soil or water beyond a threshold that inhibits plant growth [3].

In plants, salt exposure generates osmotic stress and ion cytotoxicity, impairs seed germination and shoot development, enhances leaf senescence, and inhibits major cellular processes such as protein and lipid synthesis, photosynthesis, and energy metabolism [4,5]. To alleviate salt stress, plants must create intricate systems that incorporate complex signaling networks [6]. These mechanisms activate the plant stress hormone abscisic acid (ABA), sense cellular nourishment, and regulate protein changes [7].

Plant growth regulators such as melatonin have been explicitly reported to mitigate the phytotoxicity induced by various biotic and abiotic stresses [8]. Melatonin biosynthesis in crops begins with tryptophan as a precursor, and is regulated by the genes *TPH*, *T5H*, *TDC*, *ASMT*, *SNAT*, and *COMT,* which together regulate two major pathways: (i) the tryptophan/tryptamine/serotonin/*N*-acetylserotonin/melatonin pathway, and (ii) the tryptophan/tryptamine/serotonin/5-methoxytryptamine/melatonin pathway [9]. Melatonin plays a crucial role in ionic balance, scavenging toxic radicals, increasing antioxidant enzyme activity, inhibiting active oxygen explosion, reducing malondialdehyde (MDA) content and relative conductivity, and improving photosynthetic attributes to confer tolerance during stressed conditions in plants [10,11]. Melatonin synthesis in crops is mostly determined by the concentration of metal ions, hydroxyl radicals, and environmental variables that control the enzymatic reaction pathways of tryptophan, tryptamine, and serotonin [12].

Additionally, a monosaccharide and a monomeric unit of the polymer chitin, namely N-acetylglucosamine (GlcNAc) [13,14], has been reported to regulate various functionalities in plant physiology such as cell signaling, pathogenesis, immune responses, and activation of defense-related genes via protein glycosylation [15,16]. Protein glycosylation impairment may lead to several phenotypic variations as demonstrated by Jia et al. [17] in Arabidopsis. Hence, understanding the metabolomics of crops given any treatment under stress is crucial to cross-check both the pros and cons. For these to resolve, parameters such as optimum deposition of minerals, ion regulation, amino acid programming, antioxidant activation, radical scavenging, phytohormone synthesis, and photosynthetic components determine some of the major functional roles [18].

The cucumber, or *Cucumis sativus* L., from the Cucurbitaceae family, is a popular vegetable. It is traditionally employed in a variety of treatments and demonstrates a number of medical qualities, including antibacterial activity, the ability to decrease blood sugar, antioxidant capacity, etc. [19]. A growth regulator plays a unique role in plant metabolism and improving the nutritional status of crops such as cucumber [20]. The JIP test is one of the most successful and widely used methods for studying the behavior of the photosynthetic apparatus, specifically the O-J-I-P kinetic transient fluorescence. It determines several phenomenological and biophysical expressions such as the activation of the reaction center, light absorption, electron transfer, heat dissipation, etc. that quantify the function of PSII [21,22,23]. In the current study, we treated GlcNAc and Mel simultaneously in cucumber plants and observed their effect on various metabolic aspects and photosynthetic traits using the OJIP test. It was predicted that the N-acetylglucosamine and melatonin combined application would exert a synergistic effect to reduce oxidative stress caused by salinity. To the best of our knowledge, this is the first report to demonstrate the interaction of GlcNAc/Mel in regulating the photosynthetic attributes and metabolomics of the cucumber plant.

## 2. Results

### 2.1. Evaluation of Morphological Attributes of Cucumber Plants

**Figure 1:**
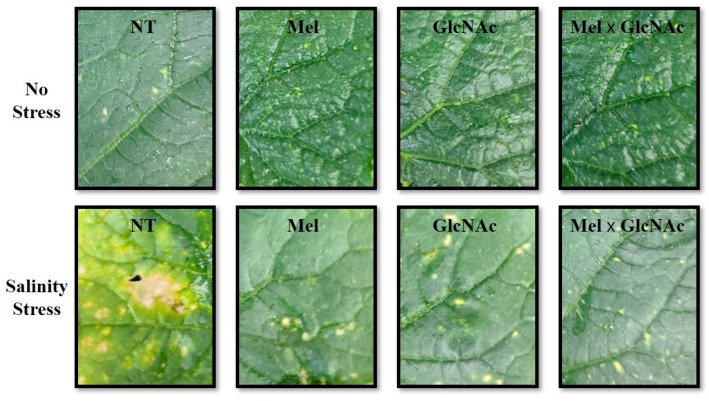
Visual observation of the effect of Mel/GlcNAc in the leaves of Cucumber plants.

It was observed that the Mel × GlcNAc treatment significantly increased the shoot length, root length, shoot fresh weight, root fresh weight, shoot dry weight, root dry weight, and stem diameter by 20%, 17%, 31%, 27%, 16%, 30%, and 12%, respectively, under NS when compared to NT. A similar trend was observed under SS where Mel × GlcNAc significantly increased the SL, RL, SFW, RFW, SDW, RDW, and SD by 30%, 39%, 45%, 51%, 46%, 56%, and 23.9%, respectively, when compared to NT. Salinity stress induced necrosis in crop leaves, which was significantly improved with the treatment of GlcNAC and Mel (Figure 1). The combined treatment of Mel × GlcNAc was observed to be efficient in the improvement of plant visual appearance and morphological traits when compared to NT or sole application. The sole application of Mel or GlcNAc also considerably improved the overall plant growth-promoting traits (Table 1). 

### 2.2. Extraction and Quantification of Abscisic Acid and Measurement of Leaf Water Content

**Figure 2:**
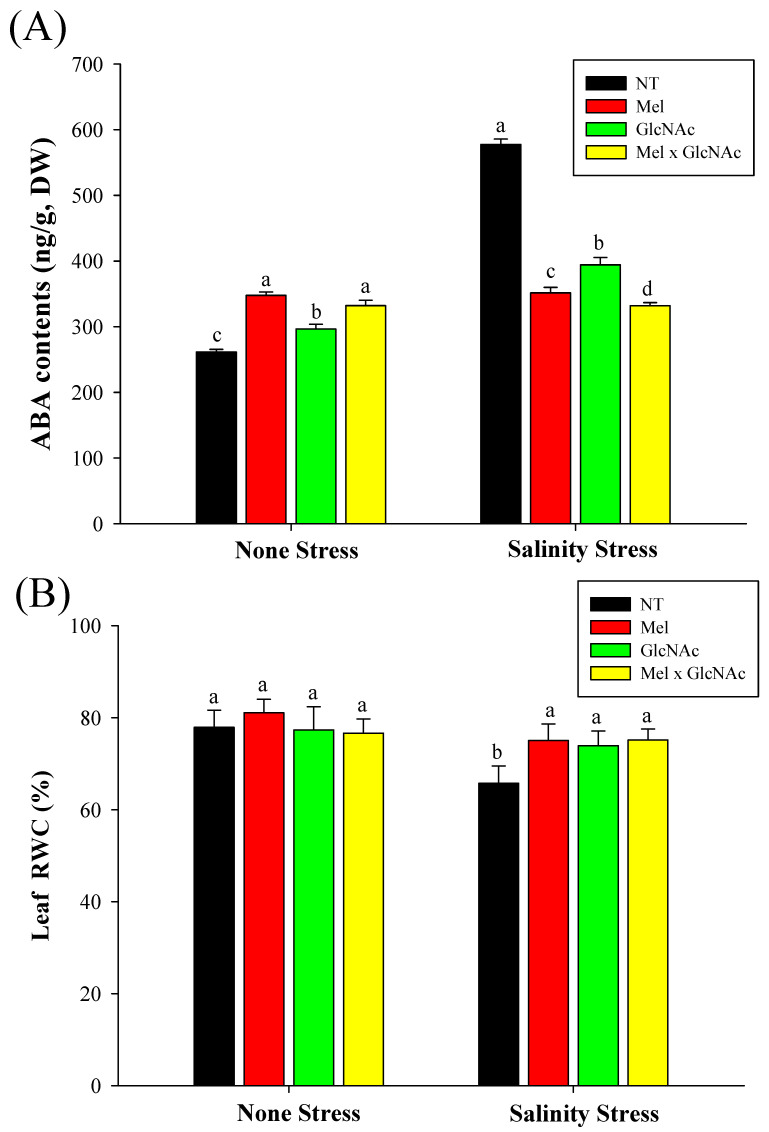
Quantification of abscisic acid level (**A**) and relative water content (**B**) in cucumber leaves. Error bars represent the mean ± standard deviation. Each data point represents the mean of at least three replicates. Bars with different letters are significantly different at *p* ≤ 0.05. The abbreviations used in the treatments are detailed in Table 1.

The ABA content was increased by 33%, 13%, and 27%, respectively, with Mel, GlcNAc, and Mel × GlcNAc treatment under NS. However, Mel, GlcNAc, and Mel × GlcNAc treatment significantly dropped the ABA content by 39%, 31%, and 43%, respectively, under the SS condition. Moreover, it was observed that the leaf RWC content was significantly dropped under NT by 15% under SS when compared to the control under NS. The RWC was increased by 14%, 12%, and 14% after treatment with Mel, GlcNAc, and Mel × GlcNAc, respectively, under SS. No differences were observed in RWC in the NS condition (Figure 2).

### 2.3. Quantification of Amino Acids and MDA Content in Cucumber Shoots

**Figure 3:**
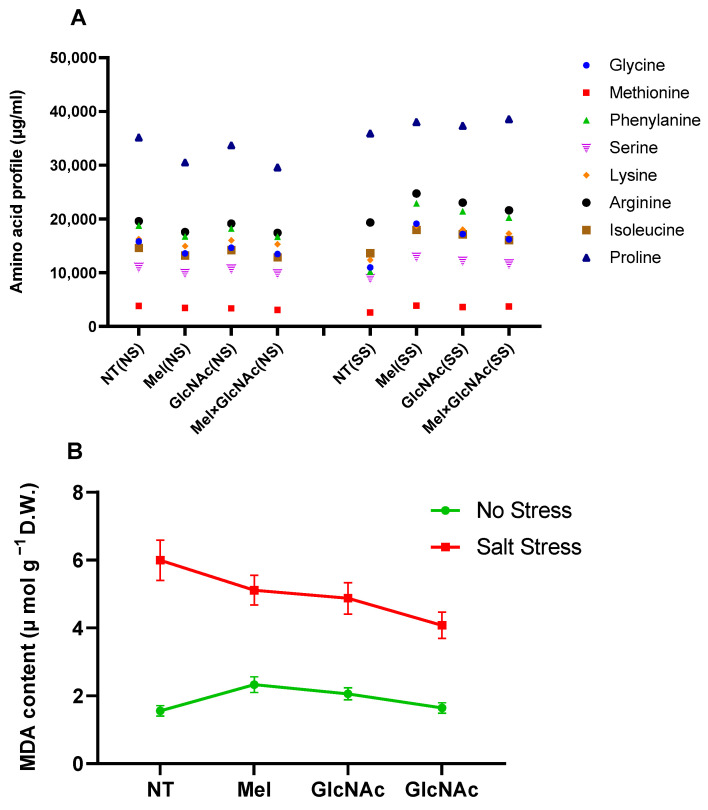
Amino acid profile (**A**) and malondialdehyde (**B**) quantified in cucumber leaves under normal and stressed conditions. Each data point represents the mean of at least three replicates (mean ± standard deviation). NS: No Stress, SS: Salt Stress.

In affected plants, the salinity stress condition considerably dropped the synthesis of all amino acids, such as glycine, methionine, phenylalanine, serine, lysine, arginine, isoleucine, and proline, indicating a higher protein degradation rate. However, only a minor fluctuation was observed in synthesis when the treatment of Mel/GlcNAc was applied, in all cases. Additionally, the lipid peroxidation rate was significantly elevated by salt stress, whereas Mel/GlcNAc treatment significantly downregulated the MDA generation under salt stress when compared to NT (Figure 3).

### 2.4. Determination of Chlorophyll, Chlorophyll Fluorescence Ratio, and Relative Water Content

**Figure 4:**
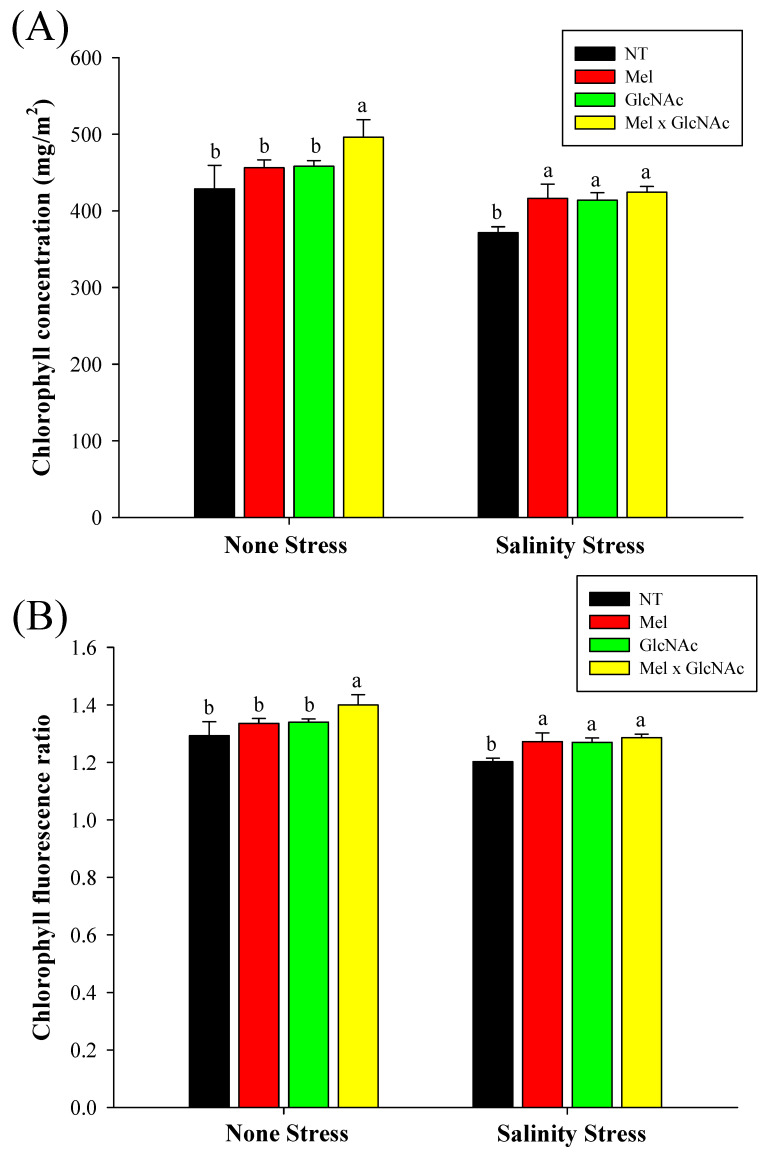
Measurement of chlorophyll concentration (**A**) and chlorophyll fluorescence (**B**) in cucumber leaves. Error bars represent the mean ± standard deviation. Each data point represents the mean of at least six replicates. Bars with different letters are significantly different at *p* ≤ 0.05. The abbreviations used in the treatments are detailed in Table 1.

The chlorophyll content was significantly increased by Mel × GlcNAc, by 16% and 14% under NS and SS, respectively. The sole treatments of Mel and GlcNAC increased chlorophyll content by 12% and 11% under SS, respectively, whereas minor differences of 6% and 7% was observed under NS. The chlorophyll fluorescence ratio was increased to 1.28 with Mel × GlcNAc treatment when compared to NT (1.2) under NS. However, under stress, Mel, GlcNAc, and Mel × GlcNAc all increased the CFR ratio, to 1.27, 1.27, and 1.28, respectively, when compared to NT (1.2) (Figure 4).

### 2.5. OJIP Curve and Test Parameters

**Figure 5:**
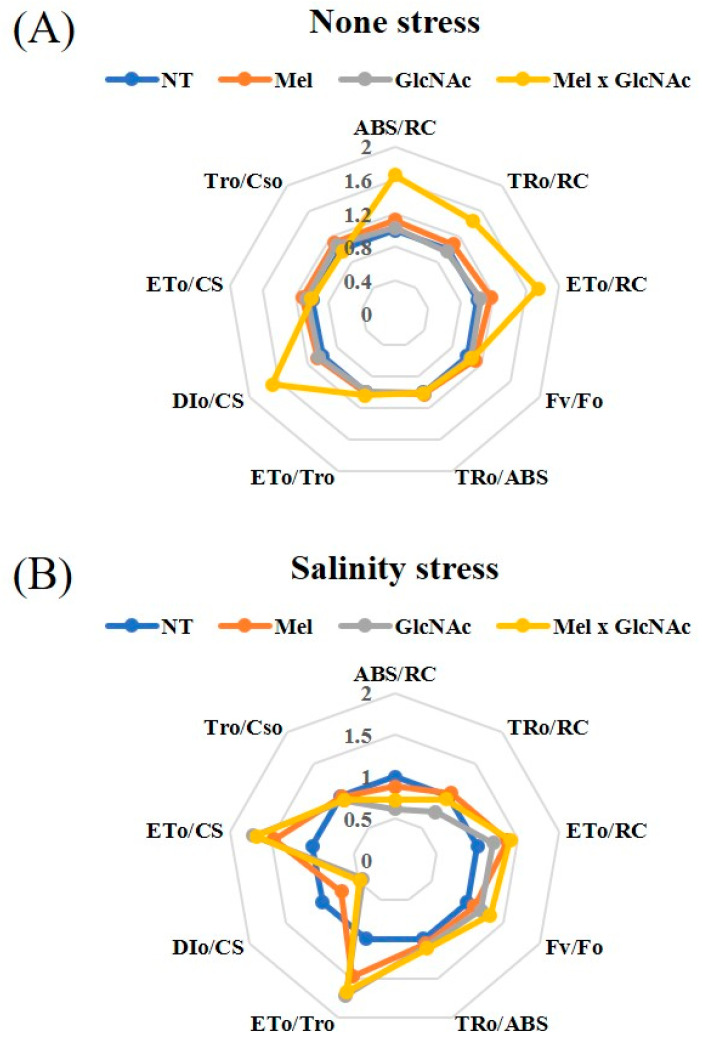
Illustration of OJIP parameters of transient chlorophyll fluorescence using radar plot under Normal (**A**) and Salt Stressed (**B**) condition. The abbreviations are detailed in Table 2.

The trends of ABS/RC, TRo/RC, ETo/RC, Fv/Fo, TRo/ABS, ETo/TRo, and DIo/CS are considerably increased by all the treatments, with maximum values observed with Mel × GlcNAc in DIo/CS, and ABS/RC and Eto/RC under NS. The ETo/CS and TRo/CSo were dropped with treatment using Mel × GlcNAc under NS. The absorption and trapping flux (ABS/RC and TRo/RC) were reduced under SS, indicating the deactivation of the reaction centrals. The Mel × GlcNAc treatments recovered several of the components under stress, enhancing electron transport and the quantum yield. The minimum value was observed at Dio/CS and Tro/CSo, and showed only minor or no differences in all conditions when compared to NT (Figure 5).

The reduction in Fv/Fm value is due to the lower passage of electrons and photoinhibition of PSII that generally indicates deactivation of reaction centrals. FV/Fo value determines the specific flow of energy dissipation per active reaction center (Eto/RC). Apart from the treatments, the intensity of light and diffraction plays a crucial role in the activation/deactivation of the photosystem and electron transport system. Cao et al. [24] demonstrated this phenomena with the effect of plant nitrogen in *Lonicera japonica*, where the increase in light absorption per active reaction central (*ABS*/*RC*) accompanied by an increase in light trapping per active reaction central (*TR*o/*RC*) drove an increase in electron transport flux (*ET*o/*RC*). These led to enhanced efficiency of electron transfer to plastoquinone (*ET*o/*TR*o), simultaneously increasing the photochemistry efficiency (*TR*o/*ABS*) and ultimately increasing the heat dissipation ratio (*DI*o/*CS*).

### 2.6. Measurement of Photosynthetic Rate (Pn), Stomatal Conductance (Gs), Transpiration Rate (E), and Substomatal CO_2_ Concentration

**Figure 6:**
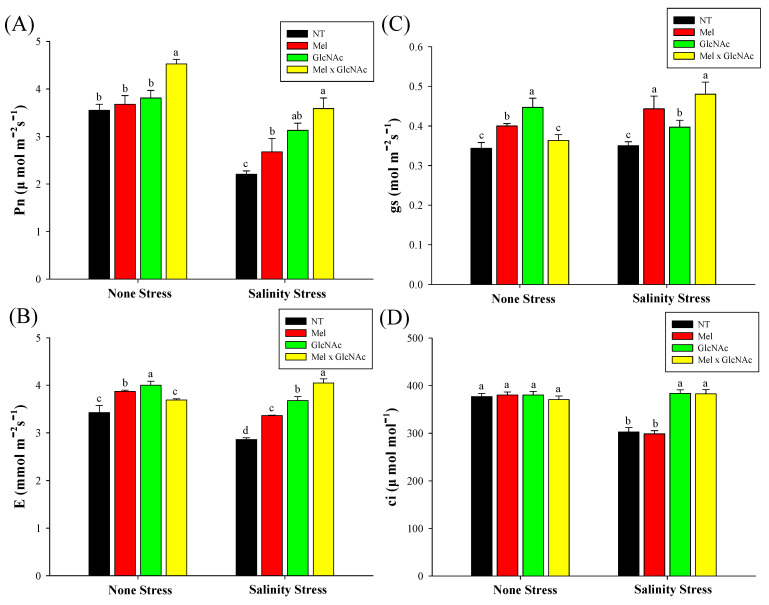
Photosynthetic rate—Pn (**A**), transpiration rate—E (**B**), stomatal conductance—Gs (**C**), and substomatal CO_2_ concentration—Ci (**D**) of cucumber leaves treated with Mel/GlcNAc under salt stress. Error bars represent the mean ± standard deviation. Each data point represents the mean of at least six replicates. Bars with different letters are significantly different at *p* ≤ 0.05. The abbreviations used in the treatments are detailed in Table 1.

The Pn was significantly increased by Mel × GlcNAc under NS, whereas sole application showed only minor or no differences. Similarly, Gs, E, and Ci levels also showed minor differences under NS in all treatments except GlcNAc(gs). Under SS, the Pn was increased by 21%, 42%, and 62%, Gs was increased by 25%, 11%, and 37%, and E was increased by 17%, 28%, and 41%, with Mel, GlcNAc, and Mel × GlcNAc, respectively. The Ci was increased by 27% and 26% with GlcNAc and Mel × GlcNAc, respectively, under SS, while Mel treatment significantly reduced Ci by 21% when compared to NT (Figure 6).

### 2.7. Quantification of K^+^, Mg^+^, Na^+^, Ca^++^ and P Content in Cucumber Shoots

The ICP analysis showed that the K/Na ratio showed only minor differences with Mel, GlcNAc, and Mel × GlcNAc treatments under NS. The K/Na ratio, however, was increased by 62% and 95% by Mel and Mel × GlcNAc, respectively. The GlcNAc showed no effect on the K/Na ratio in SS. The K content was increased by 41% and 27% by Mel × GlcNAc in the NS and SS conditions, respectively. The Mg content was increased by 92%, 21%, and 60%, the Ca content was increased by 85%, 21%, and 56%, and the P content was increased by 48%, 7%, and 49% with Mel, GlcNAc, and Mel × GlcNAc treatment, respectively, under NS when compared to NT.

**Figure 7:**
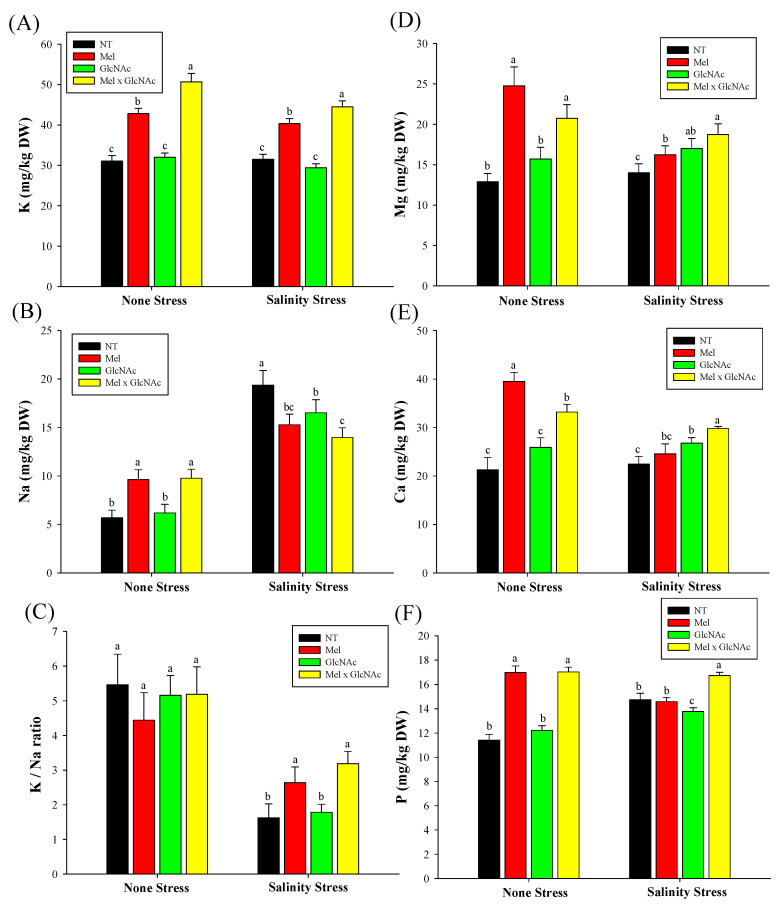
Analysis of mineral elements potassium (**A**), sodium (**B**), K/Na (**C**), magnesium (**D**), calcium (**E**), and phosphorus (**F**) in cucumber shoots. Error bars represent the mean ± standard deviation. Each data point represents the mean of at least six replicates. Bars with different letters are significantly different at *p* ≤ 0.05. The abbreviations used in the treatments are detailed in Table 1.

The Mg content was increased by 15%, 21%, and 33%, and Ca content was increased by 9%, 19%, and 32%, respectively, with Mel, GlcNAc, and Mel × GlcNAc under SS. The P content showed no differences with a sole application of Mel or GlcNAc, whereas Mel × GlcNAc increased P content by 13% under SS (Figure 7).

### 2.8. Analysis of Antioxidants and Their Related Activity

**Figure 8:**
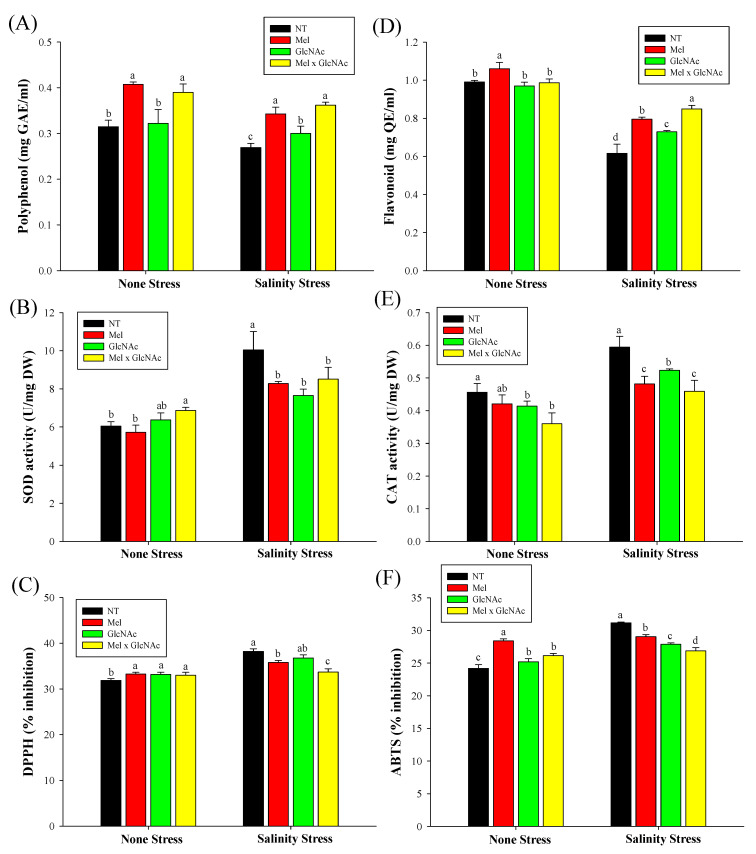
Assay of antioxidant related activity, enzymes, and radical scavengers. (**A**) Polyphenol, (**B**) superoxide dismutase, (**C**) DPPH, (**D**) flavonoid, (**E**) catalase, and (**F**) ABTS. Error bars represent the mean ± standard deviation. Each data point represents the mean of at least six replicates. Bars with different letters are significantly different at *p* ≤ 0.05. The abbreviations used in the treatments are detailed in Table 1.

The polyphenol content was increased by 27%, 11%, and 34%; Flavonoid by 29%, 18%, and 37%; SOD decreased by 17%, 23%, and 15%; and CAT decreased by 19%, 11%, and 22% with Mel, GlcNAc and Mel × GlcNAc, respectively, under SS. Under NS, Mel × GlcNAc enhanced polyphenol, SOD, and DPPH, and reduced CAT content. The DPPH and ABTS content were considerably reduced under stressed conditions and slightly increased under NS (Figure 8).

## 3. Discussion

The application of biostimulants has been recognized as an emerging tool to improve crop growth and development. Several studies have found that the use of melatonin in crops improves plant resistance by neutralizing ROS and boosting antioxidant enzyme activity, metabolites, and transcription factors. Similarly, GlcNAC has been reported as a potent regulator of plant physiology; nevertheless, the research with regard to GlcNAC application in plant abiotic stress is limited. Here, we described the possible mechanism of GlcNAC and melatonin interaction in regulating the photosynthetic attributes and metabolomic aspects of cucumber plants exposed tosalt stress.

In salt-stressed plants, the toxicity generated by the increased buildup of Na^+^ and Cl^-^ and the outflow of K+ is a critical event [25]. In our experiments, the Na^+^ content was significantly elevated under SS in cucumber plants. However, the GlcNAc/Mel treatment greatly elevated the Ca^2+^ and K^+^ intake and lowered the Na^+^ influx in salt-stressed cucumber plants. Plant salt tolerance is directly related to the capacity to maintain an appropriate Na^+^/K^+^ ratio [26]. The optimum balance of mineral ions is crucial to combat oxidative stress and enhance the efflux of toxic radicals. For this to happen, the ion supply route of a plant needs stability to undergo the influx/efflux phenomena. Supplements of exogenous minerals such as Ca, K, Mg, and P have often been recommended to enhance ionic balance in crops under several abiotic stresses, including salinity [27]. In addition to ion supplementation, the application of a growth regulator could play a crucial role in the osmotic balance of the cell, as observed in our findings with the application of Mel and GlcNAC. These phenomena in general are greatly regulated by endogenous phytohormones, namely abscisic acid, antioxidants (CAT, SOD), radical scavengers, and amino acids.

The increase in mineral elements under treatment with GlcNAc/Mel was also observed in the NS condition, and simultaneously, an elevated level of ABA was observed. Since an elevation in ABA is directly related to stomatal conductance and water uptake, this plays a significant role in ion balance, followed by enhanced mineral nutrient uptake. These would not only enhance the resistance of crops against abiotic stress, but also several biotic stresses. These phenomena include the cross-talk between hormones such as salicylic acid, jasmonic acid, and ethylene that regulates pathogen-associated molecular patterns (PAMPs) and activaties PAMP-triggered immunity [28].

The interrelationships of abscisic acid and proline content in water balance and electrolytic leakage to regulate stomatal conductance have been widely accepted by several researchers [29]. ABA plays a crucial role in the sustenance and degradation of proline synthesis, particularly in water-deficient conditions where proline directly regulates the guard cell movement, stomatal conductance, and ultimately transpiration rate triggered by ABA [30]. In the current findings, we observed that proline and ABA synthesis showed a vice-versa association. The Mel × GlcNAc treatment dropped the ABA level under stress, meanwhile increasing the proline content. Our results are in line with the findings of Zhang et al. [31], who demonstrated a possible phenomenon suggesting that the melatonin triggered abscisic acid (ABA) catabolism during seed germination of cucumber by downregulating the ABA biosynthesis genes (*CsNECD2*) and upregulating ABA catabolism genes (*CsCYP707A1* and *CsCYP707A2*). The higher proline content in the cucumber plants created an enhanced adaptive mechanism under salinity, which is attributed to the findings of Sarropoulou et al. [32]. Similar results were shown by Dawood and El-Awasdi [33] and Han et al. [34], who showed that melatonin treatment increased proline, thereby enhancing the defense mechanism of *Vicia faba* and rice plants under salt and cold stress, respectively [35]. These findings were further validated by the increases observed in stomatal conductance (Gs), transpiration rate (E), substomatal CO_2_ concentration (Ci), and leaf water content. The osmotic balance sustained by ion and water balance is modulated by stress hormones, such as ABA, and amino acids, mainly proline, and further strengthens the antioxidant system to scavenge toxic oxygen radicals.

ROS are the typical free radical that cause toxicity in response to all stimuli. Biological activities like growth, the cell cycle, programmed cell death, and signaling are all regulated and controlled by ROS [36]. These reactive species are produced in chloroplasts, mitochondria, peroxisomes, apoplasts, plasma membranes, the endoplasmic reticulum, and cell walls, and result in oxidative damage to protein, lipids, and DNA. They also disrupt photosynthesis, causing tissue necrosis and cell death [37,38]. The antioxidant enzymes SOD and CAT play a dynamic role in neutralizing ROS. SOD converts O_2_ into H_2_O_2_, serving as the initial line of defense against ROS. APX, GPX, and CAT then further detoxify the resultant H_2_O_2_ [39]. Catalases are particularly efficient in H_2_O_2_ removal and have the unique capacity to transform 2H_2_O_2_ to O_2_ + 2H_2_O [40]. Our results align with the findings of Bhardwaj et al. [41], who showed that melatonin alleviated Cd and NaCl toxicity by balancing redox homeostasis through utilizing SOD, APX, glutathione, and catalase, thereby maximizing the quantum energy (F_V_/F_M_) and performance index (PI_ABS_) in tomato.

Moreover, phenolic chemicals have been demonstrated to be beneficial in protecting biological systems from various oxidative stresses, playing a critical role in maintaining redox-homeostasis and suggesting a possible target for enhancing stress tolerance in plants [42]. Our results showed a considerable rise in polyphenol and phenolic content, thereby reducing the DPPH and ABTS content, with Mel × GlcNAc treatments. Similar results were shown by Kiani et al. [43], where salt stress increased proline content, electrolyte leakage, phenolic content, DPPH scavenging, and flavonoid in wheat.

The interrelation of antioxidants, hormones, ions, enzymes, and amino acids ultimately plays a crucial role in the photosynthesis of crops. NaCl exposure in general hampers the photosynthetic system via chlorophyll degradation, increasing lipid peroxidation and halting PSII activity [44]. During this process, a decrease in Fv/Fo may account for the fluctuation of light reactions and photochemistry [45,46], which leads to increased energy dissipation in the reaction center (Dio/RC). This dissipation of energy is then utilized for protection against photo-oxidative damage, to combat nutrient deficiency, to convert excess energy into heat, and to direct the energy to the electron transport chain (ETo/RC) [22,46].

Here we identified several photosynthetic components in which we observed that chlorophyll degradation by salt stress was recovered by Mel × GlcNAc treatment. It was observed that the chlorophyll fluorescence and several photosynthetic components such as light absorption, activation of reaction center, electron transport flux, quantum yield, and heat dissipation considerably improved with Mel × GlcNAc treatment. Our results are aligned with the findings of Ayyaz et al. [47], who reported a decrease in ratios of fluorescence (Fv/Fm, Fv/Fo) in electron transport fluxes, which caused damage to the number of active reaction centers (RC) due to chromium stress; however, they found that melatonin improved PIABS and electron transport per reaction center (ET/RC) in canola. Similar findings have been reported by a number of authors [48,49,50,51], who demonstrated that melatonin treatment restored chlorophyll fluorescence, increased net photosynthetic rate, alleviated oxidative damage, and improved the quantum yield of PSI and PSII in drought- and salinity-induced osmotic stress.

GlcNAc plays an important role in protein modification, with protein N-glycosylation being one of the most common modifications, which is crucial for plant growth and stress responses [52,53]. N-glycosylation begins with the presence of GlcNAc, an important amino sugar moiety that commences the processing of oligosaccharide precursors at the cytosolic side of the endoplasmic reticulum. Defects in N-glycan digestion frequently impact plant development and stress responses, resulting in mortality. The growth improvement of cucumber plants in our study aligned with the findings of Chen et al. [54], who reported the exogenous application of GlcNAc conferred tolerance and improved growth of wild-type Arabidopsis seedlings under salt stress conditions. Similarly, Sun et al. [55] reported that the GlcNAc treatment enhanced the growth of tomato plants via stimulation of microorganism community structure.

The synergistic potential of melatonin has been demonstrated by several authors such as Bian et al. [56], who treated *Vicia faba* with GABA + melatonin in response to different stressors (salinity (NaCl), polyethylene glycol (PEG), and sulfur dioxide (SO_2_)) and found that it significantly improved stomatal anatomy and the photosynthetic process [57]. Since GlcNAc exerts a positive impact on the cucumber plant under salt stress, the combination of GlcNAc/Mel could be one effective method to combat abiotic stresses. Additionally, NaCl stress although exerted negative effects could be an efficient elicitor of melatonin synthesis, as observed in grapevine leaves and St. John’s wort [29,56]. This could potentially be employed in the melatonin extraction process for industrial purposes.

## 4. Material and Methods

### 4.1. Plant Experiments

Cucumber (*Cucumis sativus* L.) seeds were purchased from Danong Co., Namyangju, South Korea. The seeds were surface sterilized with 5% NaOCl for five minutes before being completely rinsed with autoclaved distilled water. Seeds were sown in a 72-hole germination tray under controlled greenhouse conditions at 25 ± 2 °C. Two-week old seedlings were then transplanted to a pot (10 cm deep and 10 cm wide at the top). After a week, the seedlings were treated with 100 µM MT and 1 mM GlcNAc, enough to form water droplets on the leaves for three days, and were subjected to treatment with NaCl (150 mM; 50 mL) for five days. The plants were then normally irrigated for a week, harvested in liquid N_2_, and stored at −80 °C to perform biochemical analysis. The experiment was divided into two groups: i. NS (no stress) and ii. SS (salt stress), with each group containing the four treatments (NT: no treatment, Mel: melatonin only, GlcNAc: N-Acetylglucosamine only, and GlcNAc × Mel).

### 4.2. Measurement of Chlorophyll Fluorescence

A chlorophyll content meter (CCM300, Optisciences, ADC BioScientific, Ltd., Herts, UK) was used to measure the chlorophyll content. A chlorophyll fluorometer (Os5p Optisciences, Inc, Hudson, NY, USA) and photosynthesis meter (LCproTADC) were used to measure the photosynthetic component used for the OJIP curve. The equation described by Mora et al. [22] and Singh et al. [58] was used to calibrate the various specific energy fluxes and phenomenological energy fluxes. The test parameters are detailed in Table 2.

### 4.3. Quantification of K/Mg/Na/Ca/P

The quantification of mineral elements was performed using the method described by Adhikari et al. [59]. The freeze-dried sample (0.5 g) was extracted with 70% HNO_3_ on a heating plate for 1.5 h at 110 °C. The solvent was then recovered by passing through H_2_O_2_, utilizing Ultrawave microwave digestion equipment (milestone). After separating and filtering the supernatant, 3 mL of the extract was diluted with deionized water to a final volume of 30 mL. The final solution was measured by injection with inductive coupled plasma mass spectrometry (ICP-MS; Optima 7900DV, PerkinElmer, Akron, OH, USA).

### 4.4. Analysis of Antioxidant Activity and Enzymes

#### 4.4.1. SOD and CAT Assay and Quantification of Total Flavonoids and Phenolic Content

An OxiTec™ SOD Assay Kit biomax and an OxiTec™ Catalase Assay Kit biomax were used to quantify the SOD and CAT content of cucumber leaves. The total phenolic content was determined using an upgraded version of a previously described assay that Slinkard and Singleton [60] that employed using the Folin-Ciocalteu reagent. The phenolic levels of all samples detected at 765 nm were expressed in gallic acid equivalents (μg/mL). The analysis approach for the blank (DMSO) was identical to that used for the samples. The dilution factor was considered for all diluted samples.

The total flavonoids were analyzed by the assay reported by Cao et al. [61], with slight modification. In brief, one gram of sample was extracted with absolute methanol, followed by subsequent stepwise reaction with 5% NaNO_2_ solution, 5% Al(NO_3_)_3_, and 4% NaOH solution. The absorbance was determined at 510 nm (Thermostat, Waltham, MA, USA). The calibration was determined using the standard curve obtained from 25 to 1000 mg/L of quercetin (equivalents per milligram of dry mass of the culture samples).

#### 4.4.2. Measurement of Radical Scavenging Activity (DPPH, and ABTS)

The DPPH scavenging activity was ascertained using the detailed protocol mentioned by Gulati et al. [62]. In brief, a volume of 50 μL of plant extract (range 20 to 1000 μg/mL) was combined with 150 μL of 0.1 mM DPPH in methanol and left at room temperature for 60 min in the dark. At 490 nm, the absorbance was measured following incubation. Plant extracts were replaced with 50 μL of ethanol in the control group, and the outcomes were compared. Ascorbic acid and butyl hydroxyl toluene (BHT) in concentration ranges of 3.12 to 250 μg/mL served as the positive controls. The percentage (%) suppression of the antioxidant activity was calculated as follows: absorbance of the control (490 nm − absorbance of samples (490 nm)/absorbance of the control (490 nm) × 100.

The ABTS cation scavenging activity was determined using 2,2-azinobis (3-ethylbenzthiazoline-6-sulphonic acid), as reported by Saeed et al. [63]. In brief, ABTS solution (7 mM) was reacted with potassium persulfate (2.45 mM) solution and left overnight in the dark to produce a dark-colored solution containing ABTS radical cation. To prepare for the test, the ABTS radical cation was diluted with 50% methanol to achieve an Initial absorbance of 0.70 ± 0.02 at 745 nm. The temperature was set to 30 °C. To analyze free radical scavenging activity, 300 μL of test material was mixed with 3.0 mL of ABTS working standard in a microcuvette. The % inhibition was estimated using the formula:

Percentage inhibition (%) = [1 − (absorbance of sample at 475 nm/absorbance of control at 475 nm)] × 100%.

The anti-radical activity, or EC50, of test samples was used to express their antioxidant capacity. This is the dose required to reduce ABTS by 50%.

### 4.5. Extraction and Quantification of Abscisic Acid

The method described by Kang et al. [64] was followed for the quantification of ABA. The values were quantified through GCMS (6890 N network GC system and 5973 network mass selective detector, Agilent Technologies, Palo Alto, CA, USA). [(±)-3,5,5,7,7,7-d^6^]-ABA was used as an standard reference. Lab-Base (ThermoQuset, Manchester, UK) data system software (G1701DA Version D.00.00.38, 19 Nov-2001) was used to monitor responses to ions of m/e (162 and 190) and *m*/*z* (166 and 194) for Me-[_2_H^6^]-ABA.

### 4.6. Analysis of Amino Acids Profile in Cucumber Shoots

The amino acid content was extracted and quantified according to the method described by Bhatta et al. [65]. Freeze-dried plant samples were hydrolyzed in 6 N HCl under vacuum in 4 mL tubes at 110 °C for 24 h, then at 80 °C for 24 h. The dried residue was homogenized with 0.02 N HCl and passed through a 0.45-μm filter. Amino acids were measured using an automatic analyzer (Corporation L-8900, Tokyo, Japan) connected to a HITACHI HPLC system (packed column with ion-exchanging resin, No. 2622 PF; 4.6 × 60 mm) and UV detector (VIS1: 570 nm, VIS2: 440 nm).

### 4.7. Statistical Analysis

The experimental analysis was carried out in triplicate. The mean values were compared using Duncan’s multiple range test (DMRT) with a significance threshold of 0.05. The Statistical Analysis System (SAS 9.1) was used for the DMRT analysis. The mean and standard deviation were calculated using Microsoft Excel 2019, and the data were graphed using SigmaPlot 10.0 software (Systat Software, Inc., San Jose, CA, USA) and GraphPad Prism (Graph Pad Software Inc., Version 8; San Diego, CA, USA).

## 5. Conclusions

Melatonin and GlcNAc co-application plays a vital role in modeling the plant metabolism and exerting beneficial effects to some extent. However, it is important to note that research in this area is ongoing, and the specific effects of this co-application may vary depending on factors such as concentration, timing of application, and the severity of salinity stress. Hence, the efficacy of these compounds can be influenced by the plant species and its developmental stage. Additionally, since melatonin and GlcNAc both play a pivotal role in animals and are explicitly used in pharmaceuticals, the current findings could bridge the gap between agriculture and pharmacology. Although the interaction of GlcNAc × Mel exerted a beneficial effect on cucumber plants in controlled conditions, further experiments are required to support field application.

## Figures and Tables

**Table 1:** Effect of sole or combined application of Mel/GlcNAc on the morphological traits of cucumber plants. Each data point represents the mean of at least six replicates (mean ± standard deviation). The column with different letters are significantly different from each other at *p* ≤ 0.05. SL: Shoot length. RL: Root length. SFW: Shoot fresh weight. RFW: Root fresh weight. SDW: Shoot dry weight. RDW: Root dry weight. SD: Stem diameter.

	SL (cm)	RL (cm)	SFW (g)	RFW (g)	SDW (g)	RDW (g)	SD (mm)
	**No Stress (NS)**						
NT	23.9 ± 0.59 b	22.9 ± 0.22 b	13.16 ± 0.35 b	5.20 ± 0.13 b	1.68 ± 0.05 c	0.40 ± 0.02 c	7.12 ± 0.03 b
Mel	26.2 ± 0.30 a	24.5 ± 0.44 ab	16.33 ± 0.29 a	5.88 ± 0.15 ab	1.89 ± 0.03 b	0.48 ± 0.01 b	7.22 ± 0.10 b
GlcNAc	27.0 ± 0.54 a	24.8 ± 0.29 ab	16.82 ± 0.30 a	5.91 ± 0.16 ab	1.84 ± 0.04 b	0.46 ± 0.02 b	7.79 ± 0.07 a
Mel × GlcNAc	28.7 ± 0.20 a	26.9 ± 0.33 a	17.30 ± 0.17 a	6.64 ± 0.20 a	1.96 ± 0.02 a	0.52 ± 0.02 a	7.96 ± 0.03 a
	**Salinity Stress (SS)**						
NT	18.9 ± 0.43 b	18.0 ± 0.34 c	9.40 ± 0.57 c	4.15 ± 0.37 c	1.21 ± 0.06 c	0.32 ± 0.02 c	6.31 ± 0.09 c
Mel	20.6 ± 0.70 b	22.1 ± 0.41 b	10.34 ± 0.37 b	6.21 ± 0.40 a	1.59 ± 0.07 b	0.44 ± 0.02 b	7.03 ± 0.06 b
GlcNAc	20.8 ± 0.55 b	22.8 ± 0.47 b	11.41 ± 0.47 b	5.76 ± 0.37 b	1.49 ± 0.06 b	0.45 ± 0.02 b	7.06 ± 0.05 b
Mel × GlcNAc	24.6 ± 0.44 a	25.1 ± 0.38 a	13.63 ± 0.26 a	6.30 ± 0.29 a	1.77 ± 0.02 a	0.50 ± 0.03 a	7.82 ± 0.14 a

**Table 2:** Abbreviations of test parameters from radar plot.

ABS/RC	Absorption Flux per RC
**TRo/RC**	Trapping flux per RC
**ETo/RC**	Electron transport flux per RC
**Fv/Fo**	Ratio of variable fluorescence and minimum fluorescence
**TRo/ABS**	Maximum quantum yield of the apparatus primary photochemical
**ETo/TRo**	Electron movement efficiency
**DIo/CS**	Dissipated energy flux per CS (at *t* = 0)
**ETo/CS**	Electron transport flux (further than QA−) per RC
**TRo/CSo**	Trapping flux (leading to QA reduction) per Cso

## Data Availability

The data will be made available upon request.
